# Current Arboviral Threats and Their Potential Vectors in Thailand

**DOI:** 10.3390/pathogens10010080

**Published:** 2021-01-18

**Authors:** Chadchalerm Raksakoon, Rutcharin Potiwat

**Affiliations:** 1 Department of Chemistry, Faculty of Science, Kasetsart University, Bangkok 10900, Thailand; fsciclr@ku.ac.th; 2 Department of Medical Entomology, Faculty of Tropical Medicine, Mahidol University, Bangkok 10400, Thailand

**Keywords:** emerging infectious diseases, arboviruses, vector, *Aedes* spp., veterinary

## Abstract

Arthropod-borne viral diseases (arboviruses) are a public-health concern in many regions of the world, including Thailand. This review describes the potential vectors and important human and/or veterinary arboviruses in Thailand. The medically important arboviruses affect humans, while veterinary arboviruses affect livestock and the economy. The main vectors described are mosquitoes, but other arthropods have been reported. Important mosquito-borne arboviruses are transmitted mainly by members of the genus *Aedes* (e.g., dengue, chikungunya, and Zika virus) and *Culex* (e.g., Japanese encephalitis, Tembusu and West Nile virus). While mosquitoes are important vectors, arboviruses are transmitted via other vectors, such as sand flies, ticks, cimicids (Family *Cimicidae*) and *Culicoides*. Veterinary arboviruses are reported in this review, e.g., duck Tembusu virus (DTMUV), Kaeng Khoi virus (KKV), and African horse sickness virus (AHSV). During arbovirus outbreaks, to target control interventions appropriately, it is critical to identify the vector(s) involved and their ecology. Knowledge of the prevalence of these viruses, and the potential for viral infections to co-circulate in mosquitoes, is also important for outbreak prediction.

## 1. Introduction

Arboviral diseases impact human and/or veterinary health in Thailand. Important vector-borne diseases affecting humans in Thailand include dengue (DENV), Zika (ZIKV), chikungunya (CHIKV), Japanese encephalitis (JEV), West Nile (WNV), leishmaniasis, malaria, and rickettsial diseases [1,2,3,4,5]. Other vector-borne viruses of lesser importance recorded from Thailand include *Tembusu virus*, *Kaeng Khoi virus* and tick-borne viruses (e.g., Langat virus) [6,7,8]. Multiple arthropod vectors have been recorded from Thailand, some not necessarily yet linked as causal agents of disease outbreaks in this country but known to be transmission agents elsewhere. These include various species of mosquitoes in the genera *Aedes*, *Anopheles*, *Mansonia* and *Culex*, and also other arthropods such as sand flies, ticks, fleas, black flies, and lice [9,10,11,12,13,14].

Blood-feeding insects of the family *Cimicidae,* which include bed bugs, such as *Cimex hemipterus* and *Cimex lectularius*, have been recorded in Thailand. These two species feed on humans but are not known to transmit the disease to humans, despite previous records of organisms such as *Coxiella burnetii* and *Wolbachia* spp. among bacteria, *Aspergillus* spp. among fungi, and hepatitis B virus and human immunodeficiency virus (HIV) among viruses recorded from cimicid specimens [15].

Recently, several arboviruses outbreaks were reported in many countries of the world. Meanwhile, vector and pathogen relationships or important arboviruses are rarely recorded in Thailand. Our aim was to summarize the important human and veterinary arboviruses and vectors reported during the period 2009–2019 and to describe the arboviruses that have since also been recorded in Thailand (Table 1).

## 2. Arboviruses and Vectors in Thailand

### 2.1. Important Human Arboviruses in Thailand

#### 2.1.1. Dengue Virus (DENV)

Dengue virus causes dengue fever (DF), dengue hemorrhagic fever (DHF) and dengue shock syndrome (DSS) annually, and the co-circulation of dengue viruses 1–4 (DENV 1–4) has been reported in Thailand [24,25]. The two most important vectors are *Ae. aegypti* and *Ae. albopictus*, and they are competent vectors of all four dengue virus serotypes in Thailand [24]. DENV-5 has been reported in Malaysia, and the vectors associated with DENV-5 transmission are *Ae. aegypti* and *Ae. albopictus*, which circulate in South East Asia, including Thailand. *Ae. aegypti* and *Ae. albopictus* have also been reported as vectors of CHIKV and ZIKV in Thailand, and coinfection of dengue, chikungunya or Zika virus has been reported among wild mosquitoes and in a laboratory setting (Table 2) [24,26,27,28,29].

Dengue virus is transmitted by *Aedes* (subgenus *Stegomyia*) mosquitoes include *Ae. aegypti*, *Ae. albopictus*, *Ae. polynesiensis*, while other members of the *Ae. scutellaris* group has long been recognized as potential vectors in the Torres Strait, Australia [30]. *Ae. scutellaris* has been found in coastal areas in Thailand, but virus transmission via this species is still unconfirmed [17]. Aside from its urban and peri-urban circulation in *Ae. aegypti* and *Ae. albopictus*, dengue virus is also maintained by Aedes vectors in sylvatic cycles, such as by *Ae. niveus* (subgenus *Finlaya*) in forests in Asia and Africa, and it has not been reported yet as a dengue virus vector in Thailand [31,32].

Transovarial transmission (TOT) is an important factor contributing to the environmental maintenance of the dengue virus in endemic areas [33]. The presence of natural vertical transmission in *Ae. aegypti* and *Ae. albopictus* was clearly evidenced in Thailand, significantly influencing the epidemiology of dengue virus transmission [24,34].

#### 2.1.2. Chikungunya Virus (CHIKV)

CHIKV is a mosquito-transmitted virus. The first reported case in Thailand occurred in 1958. After the first report, outbreaks occurred in Nongkai (northeast) and Nakornsri-thamaraj (south) in 1995 [35]. Chikungunya fever re-emerged in Thailand in 2008–2009; it was caused by the East/Central/South Africa (ECSA) genotype of the virus [36]. No drug or vaccine is effective against CHIKV. Thus, interrupting viral transmission by mosquito control remains the most effective strategy for controlling CHIKV infection.

The transmission cycles of CHIKV in Asia and Africa are distinct. In Africa, CHIKV is maintained in a sylvatic, enzootic cycle in voles to non-human primates as reservoirs or amplifying hosts, and the vectors are *Aedes* mosquitoes; *Ae. africanus*, *Ae. furcifer*, *Ae. taylori*, *Ae. luteocephalus* and *Ae. neoafricanus* [37,38]. In Asia, CHIKV is maintained through the endemic/epidemic cycle via humans as a primary host and *Ae. aegypti* as a primary vector [39]. During the outbreak in 2019, CHIKV was isolated from field-caught *Ae. aegypti* mosquitoes [40].

In Thailand, *Ae. albopictus* is a primary vector of CHIKV, while *Ae. aegypti* acts as a secondary vector [41]. Phylogenetic analysis indicates that CHIKV from *Ae. aegypti* also consist of the Indian Ocean and East/South African clades, both of which belong to the ECSA genotype (Table 2). The envelope protein mutation at E1-226 A or V of the Reunion strain or a West African strain has enhanced vector specificity, *Ae. albopictus* has a higher infection rate and dissemination rate than *Ae. aegypti* vector [42]. The E1:A226V mutation of CHIKV is present in mosquitoes from Thailand: Ubon Ratchathani, Chiang Rai, Chiang Mai, Nakhon Sawan, and Songkha provinces, while the E1: K211E mutation is found in samples from Nong Khai, Bangkok, Prachuap Khiri Khan, and Krabi Province [40].

CHIKV isolated during the 2005–2006 Indian Ocean epidemic is a novel ECSA with an alanine mutated to valine at position 226 in the E1 envelope glycoprotein gene (E1-A226V). It has been subsequently described as the Indian Ocean lineage (IOL) [42,43]. The re-emergence of CHIK fever in Thailand was caused by IOL [36]. IOL is transmitted via *Ae. aegypti* and *Ae. albopictus* mosquitoes, both of which can transmit CHIKV vertically to their F5 and F6 progenies, respectively [41]. The vertical transmission of CHIKV was accomplished in a laboratory in Thailand, and it showed that *Ae. albopictus* is more susceptible to the virus and has a greater ability to transmit it vertically than *Ae. aegypti* [41]. These findings are similar to the study of CHIKV transmission in local *Ae. albopictus* mosquitoes in America and Europe [44]. Both male and female *Ae. aegypti* were found to carry CHIKV (E1: A226V and E1: K211E) infection, with infection rates of 0.85% and 3.28%, respectively [40]. However, infection rates rely on several parameters, may change from one mosquito species to another, and depend on the viral dose, temperature, etc. [45,46].

#### 2.1.3. Zika Virus (ZIKV)

The genome of ZIKV strain CVD_06-274, isolated from the serum of an infected patient in Thailand in 2006, has been completely sequenced. Moreover, ZIKV has been exported from Thailand to Japan. A case was reported after a Japanese traveler returned from Thailand in 2014, and ZIKV was detected in the urine [47]. ZIKV circulates via two transmission cycles: an enzootic sylvatic cycle and a human cycle. The sylvatic cycle involves the virus circulating between arboreal *Aedes* spp. mosquitoes and non-human primates. The human cycle involves the virus circulating between humans and peridomestic/domestic *Aedes* spp. mosquitoes. Several mosquito genera are vectors of ZIKV, including *Aedes*, *Anopheles*, *Mansonia* and *Culex* [48]. ZIKV can be transmitted venereally between *Ae. aegypti* mosquitoes under laboratory conditions [49]. ZIKV was also found actively to infect *Ae. aegypti* and *Cx. quinquefasciatus* collected from a patient’s home in Thailand (Table 2) [14]. A laboratory study has confirmed that ZIKV can be transmitted vertically in *Ae. aegypti* and *Cx. quinquefasciatus* in Thailand [50], and *Ae. aegypti* also has presented venereal transmission [49]. Both male and female *Ae. aegypti* mosquitoes can transmit ZIKV to the F1 generation, whereas *Ae. albopictus* was unable to transmit the virus vertically in the laboratory [50]. Moreover, *Cx. quinquefasciatus* can transmit the virus vertically to the F6 generation in females and to the F2 generation in males. *Ae. aegypti* and *Cx. quinquefasciatus* are thus important vectors of ZIKV in Thailand [50], which differs from ZIKV transmission in urban environments, such as in Gabon, where *Ae. albopictus* is the major mosquito vector [51].

#### 2.1.4. Japanese Encephalitis Virus (JEV)

JEV is transmitted by infected mosquitoes and causes severe encephalitis in humans. The disease is widely distributed in areas that include northern Australia, the western Pacific, and Asia [52,53]. JEV was originally isolated in 1934 from the brain of a human fetal encephalitis case in Tokyo. The virus was first isolated from *Cx. tritaeniorhynchus* mosquitoes in 1938 [52]. JEV is endemic to Thailand, causing annual averages of 1550 and 2500 cases throughout the 1970s and 1980s, respectively [53]. Between July 2003 and August 2005, the sera and cerebrospinal fluid (CSF) of 147 patients from seven hospitals in Bangkok and Hat Yai were tested [54]. Twenty-two (15%) cases were positive for JEV and 2 (1%) positive for the dengue virus. For 22 patients with JEV infection, 10 (46%) cases were patients ≤15 years old; one 13-year-old child passed away [53].

Normally, JEV is amplified in pigs and wading Ardeid birds, such as egrets and herons. It is then transmitted via the mosquito-amplifying host: humans. Several mosquito vectors can transmit JEV, the most important of which are specified in the genera *Culex*, *Aedes* and *Anopheles* [55]. *Culex* species are the primary vector for JEV transmission. Other important vectors belonging to the Culicidae are *Cx. fuscocephala*, *Cx. gelidus*, *Cx. pipiens*, *Cx. pseudovishnui*, *Cx. tritaeniorhynchus*, *Cx. vishnui* and *Cx. quinquefasciatus*. *Aedes* vectors are *Ae. japonicus*, *Ae. togoi* and *Ae. vexans nipponii*. Competent *Anopheles* vectors of JEV are *An. annularis* and *An. vagus* [55,56,57]. Numerous vector competence studies show that *Cx. tritaeniorhynchus* is the primary vector in Asia; and *Cx. tritaeniorhynchus* is abundant in a nesting colony of Ardeid birds in Thailand [55,57,58,59]. One thousand and eighty female mosquitoes have been collected with CDC light traps and GTs in Samut Songkhram Province in the central region and Phuket Province in the southern region of Thailand. Six species in the family Culicidae were determined, namely, *Cx. gelidus*, *Cx. quinquefasciatus*, *Cx. s.g. culiciomyia*, *Cx. tritaeniorhynchus*, *Cx. vishnui complex*, and *Cx. whitmorei*. Only two pools of *Cx. quinquefasciatus* were positive for JEV infection. This is the first report of JEV isolated from *Cx. quinquefasciatus* in Thailand [23].

#### 2.1.5. West Nile Virus (WNV)

WNV is a common cause of neuroinvasive arboviral disease in humans, as a dead-end host, and the virus is transmitted via infected-mosquito to the reservoir and amplifying host [60]. Studies of WNV infection in zoos and important sites for migratory and resident birds in Thailand have been conducted. A total of 66,597 mosquitoes were collected during mosquito surveillance for avifaunal sources of WNV. The results consisted of 26 species in 8 genera. The five most abundant mosquito species in the collection were *Cx. tritaeniorhynchus* (79.3%), *Cx. vishnui* (8.2%), *Cx. sitiens* (6%), *Cx. quinquefasciatus* (3.3%), and *Anopheles peditaeniatus* (1.1%). All 1736 pools from where mosquitoes were collected were negative for the presence of WNV using reverse transcriptase PCR [16].

A laboratory comparison study of WNV transmission in Thailand by *Ochlerotatus trivittatus* (COQ.), *Cx. pipiens* (L.), and *Ae. albopictus* (Skuse) vector found that *Oc. trivittatus* (COQ.) and *Cx. pipiens* (L.) are more susceptible to infection than *Ae. albopictus* when using a virus titer of <107.0 CID50s/mL. However, *Ae. albopictus* is more susceptible than *Ochlerotatus trivittatus* (COQ.) and *Cx. pipiens* (L.) when using a higher WNV concentration of >107.0 CID50s/mL [61]. Mosquito populations captured from Nakhon Pathom and Phetchaburi provinces in central Thailand have tested negative for WNV. Hence, while the findings for WNV infection are negative, mosquitoes are active in this region [62].

#### 2.1.6. Tick-Borne Viruses (TBVs)

Ticks are known vectors of transmission for a number of infectious viral diseases that arise from wild or domestic animals and are then passed on to humans [63]. The viruses carried by ticks are also known as tick-borne viruses (TBVs). They comprise a large group of viruses that belong to two orders, nine families and at least 12 genera [64]. Most TBVs are RNA viruses, some of which cause severe diseases in humans and livestock. Flaviviruses are single-stand RNA viruses that are transmitted from variable vectors. It can divide into three groups according to its vectors: the tick-borne flaviviruses (TBFVs) group, the mosquito-borne flavivirus (MBFV) group, and the no known vectors (NKV) group [64]. The genus *flavivirus* is a large group of arboviruses able to infect many vertebrates, and they can be transmitted by mosquitos, ticks, or specific arthropods vectors.

In particular, TBFVs have been recognized and divided into the mammalian tick-borne flavivirus (M-TBFV) group and the seabird tick-borne flavivirus (S-TBFV) group. This M-TBFV group contains six of the tick-borne encephalitis (TBE) serocomplex including Kyasanur forest disease virus, Langat virus (LGTV), louping ill virus, Omsk hemorrhagic fever virus, Powassan virus, and tick-borne encephalitis virus [64]. Of all listed, only the Langat virus has been found in our country, and no case of tick-borne encephalitis has been reported in Thailand [8]. The Langat virus (isolate TP21) was originally isolated from *Ixodes granulatus* (hard ticks) from forest rats caught in Malaysia [65]. In Thailand, a strain of Langat virus (isolate T-1674) was isolated from a pool of *Hemaphysalis papuana* ticks collected from vegetation in Khao Yai National Park. Both Langat virus carrier ticks, *Ixodes granulatus* and *H. papuana* are widely distributed in Thailand [66]. Both tick species have been recorded on humans, and this may suggest that the virus may be existing in other areas of Thailand [67].

The important genera of ticks involved in arbovirus transmission include *Hemaphysalis*, *Ixodes*, and *Dermacentor* [68], Table 2. Based on GPS technology, the geographical distribution of ticks in Thailand shows the important ticks species from the variable locations: *Amblyomma*, *Aponomma*, *Boophilus*, *Dermacentor*, *Hemaphysalis*, *Rhipicephalus*, and *Ornithophysalis* [69]. The *Boophilus microplus* feeds on humans in Thailand and thus represents a potential vector for zoonosis; however, it has been more specifically associated with Seletar and Wad Medani viruses. Although other tick borne-viruses have not been clearly reported in Thailand, the vectors are widely distributed in this area.

#### 2.1.7. Phleboviruses

Sandfly fever was first clinically described by Alois Pick in 1886. It is also known as “papataci fever,” “phlebotomus fever,” or “three-day fever,” and it was caused by the bite of infected female sand flies (Diptera: Psychodidae, Phlebotomine) [70]. Sandflies are small, hairy insects 2–4 mm long; there are approximately 1000 known species, 70 of which are proven vectors of leishmaniasis.

In the Old World, the sandfly-borne phleboviruses (SBPs) have been isolated from sand flies, genus *Phlebotomus*. While those from the New World have been reported from sand flies in genus *Lutzomyia* [71,72]. Sand fly-borne phleboviruses (genus *Phlebovirus*, family Phenuiviridae, order Bunyavirales) are transmitted to humans by the bite of infected female sandflies while feeding on blood. Some Old World SBPs may cause a self-limiting febrile illness (sandfly fever) or neuro-invasive infections [73]. In the Old World, SBPs are widely distributed in Africa, the Indian subcontinent, the Middle East and the Mediterranean Basin [70,74,75]. In Mediterranean countries such as Greece, the seroprevalence of phlebovirus is high (up to 60%), especially in coastal areas of the mainland and islands [74].

*Phlebovirus* is one of four genera of the family Phenuiviridae in the order *Bunyavirales*, and consist of a large group of arboviruses transmitted not only by sand flies; they can also be transmitted by members of the *Culicoides* (biting midges), ticks, flies and mosquitoes (*Culex* spp. and *Aedes* spp.) [64,76,77]. Phleboviruses have been divided into five phylogenetically related groups; the sandfly/mosquito-borne group, the Uukuniemi group, the SFTS/heartland group, the Bhanja group, and the Kaisodi group [78]. In recently, two novel tick-borne phleboviruses (TBPVs) were discovered, causing severe illness in humans. These two novel TBPVs are a severe fever with thrombocytopenia syndrome virus (SFTSV) and heartland virus (HRTV) [79]. No record or case of SFTSV/HRTV has been reported in Thailand.

Although SBPs have not yet been clearly established in Thailand, other Phleboviruses, such as the Pasi Charoen-like virus (PCLV), have been found in field-collected *Ae. aegypti* mosquitoes from Nakhon Nayok Province, Thailand [80]. Since the Rift valley fever virus (family *Bunyaviridae*, genus *Phlebovirus*) has been reported in the field-mosquito, this suggests that the ancestral host of the Phleboviruses might be the mosquito.

#### 2.1.8. Tembusu Virus (TMUV)

TMUV is a mosquito-borne *Flavivirus*. It belongs to the Ntaya virus serogroup of the Flaviviridae family [81]. TMUV is known to infect humans, was first isolated from *Cx. tritaeniorhynchus* mosquitoes in Kuala Lumpur, Malaysia, in 1955, and they were also isolated in Chiang Mai, Thailand, in 1982 [82,83]. Vector surveillance during 1982 found TMUV in pools of *Cx. vishnui*, *Cx. tritaeniorhynchus* and *Cx. gelidus* (Table 2) in Northern Thailand [83]. TMUV impacts both human and animal health, but the cause of encephalitis is restricted to chicks or ducks; particularly, the “duck Tembusu virus” (DTMUV). DTMUV is a significant veterinary pathogen and affects duck production in China and Thailand [81]. DTMUV emerged in China in 2010 and has caused decreased egg-laying, growth retardation, and neurological signs in ducks [84]. In Thailand, a new DTMUV was identified; its clinical signs include neurological manifestations, such as the inability to stand, ataxia, and paralysis [81].

DMTUV has been detected in *Cx. tritaeniorhynchus* collected from a duck farm in Sing Buri Province, Thailand, indicating a possible role for this mosquito species in the transmission cycle of DTMUV. However, the competence of this potential vector still needs to be evaluated [85]. The competence of *Cx. vishnui* captured near Sangkhlaburi, Thailand, was evaluated by allowing the mosquito to feed on TMUV-infected chicks. *Cx. vishnui* developed a high viral titer two weeks post-infection and readily transmitted the virus to naïve chickens [86]. Normally, *Cx. quinquefasciatus* predominates in urban areas, and it is also found in wide geographic areas, especially in Southeast Asia [82].

In 2015, TMUV was isolated from *Cx. quinquefasciatus* collected from Kanchanaburi Province, Thailand. This mosquito was collected from a rice paddy field and a small chicken farm near the Veterinary and Agriculture Division, Ko Samrong Subdistrict, Mueang, Kanchanaburi Province [7]. Although TMUV has never been reported in Thai patients, a potential mosquito vector was found. Overall, these results have important zoonotic implications for humans.

### 2.2. Arboviruses of Veterinary Importance Reported in Thailand, and Their Vectors

#### 2.2.1. Kaeng Khoi Virus (KKV)

The original strain of the Kaeng Khoi virus (KKV, PSC-19) was isolated from the brain tissue of dead wrinkle-lipped free-tailed bat (*Tadarida plicata* (Buchannan)) collected from Khao Wong Khot (longitude 100,033′ E, latitude 1502′ N), Ban Mi, Lop Buri Province, Thailand in 1969 [6], and may cause infections in humans [87]. KKV is a member of the genus Orthobunyavirus, Family Peribunyaviridae, Order Bunyavirales. Since 1969, the KKV was also isolated from bat ectoparasite (*Cimicidae*) or bat bugs; *Stricticimex parvus* and *Cimex insuetus* in Thailand, and implicated bat bugs as possible vectors of this virus [6]. Although KKV was found in bat bugs, it was not detected in soft ticks (*Ornithodorus hermsi*) collected from the same area [6].

Cimicids that live and feed on bats are called “bat bugs,” which are able to feed on humans if their host is absent. However, bed bugs (humans host) and bat bugs (bats host) have a similar morphology and are difficult to differentiate with the naked eye. Since 2016, our groups have reported one of bat bugs ectoparasite in Thailand. These species, *Leptocimex inordinatus*, were collected from a limestone bat cave in Kanchanaburi Province, Thailand [88]. The *L. inordinatus* have five nymph stages, and all life phases are blood-feeding on the bats’ host. They need a blood meal for growth and molts. The mode of transmission of bacterial and viral pathogens carried by *L. inordinatus* are still evaluated (unpublished).

After the initial discovery, KKV has been reported in bat flies (*Eucampsipoda sundaica*) from China [89] and was isolated from dead bats (*Chaerephon plicata*) from Cambodia [90]. The original KKV strain (PSC-19) was isolated from bats (*T. plicata* (Buchannan)) in Thailand, while KKV strain WDBC1403 was isolated from bat flies (*E. sundaica*) in China [87,89,90], was presented the highly divergent KKV from bat flies [87]. Therefore, further research on antigenicity and pathogenicity in humans and animals is required.

#### 2.2.2. African Horse Sickness Virus (AHSV)

The African horse sickness virus (AHSV) species belong to the genus *Orbivirus* of the family Reoviridae. There are nine serotypes that affect horses, with a mortality rate as high as 95% among naïve domestic horses [91]. In populations of horses that have never been exposed to the disease, case fatality rates can reach 80–90 percent, although zebra and donkeys generally suffer much milder disease. African horse sickness is endemic in sub-Saharan Africa and is listed as a notifiable disease by the World Organization for Animal Health (OIE) because of its severity and the risk of rapid global spread [92].

AHSV is a viral disease that is transmitted to mammalian hosts by biting midges of the *Culicoides* genus *Latreille*. More than 110 species are found in Africa, and *Culicoides imicola* Kieffer is considered the principal vector of AHSV in southern Africa [93], while *C. obsoletus* is a potential vector, having been implicated in the transmission of AHSV in Spain [94]. A survey of biting midges in animal sheds, mangroves and beaches along the Andaman coastal region of southern Thailand has reported a new record of *Culicoides*, such as *C. arenicola*, *C. flavipunctatus*, *C. hui*, *C. kinari*, *C. kusaiensis*, *C. parabubalus*, *C. quatei*, *C. spiculae*, *C. pseudocordiger* and *C. tamada*. This report also updated the list of *Culicoides* in Thailand [95], but the transmission vectors are still unknown.

In 2020, there was an outbreak of African horse sickness that affected horses in Nakhon Ratchasima Province, Thailand. Horse samples were sent to the Pirbright Institute in the United Kingdom, and a private veterinarian confirmed AHS-1 in March 2020 [96]. This is the first recorded presence of AHSV in Southeast Asia, where the isolate belongs to ASHV serotype 1, closely related phylogenetically to viral isolates from South Africa [96]. These results are the first report of AHS serotype 1 in Southeast Asia and outside Africa. However, transmission vectors during outbreaks are still not clear in Thailand, but the important vector, *C. imicola*, has been identified in Thailand and neighboring countries [92,95].

Disease spread can be limited by keeping horses in stables behind fine insect netting, but even the tiniest gaps between the netting and stable walls must be filled with sealant to stop the tiny insects from squeezing through. The netting and stables must also be sprayed with a pyrethroid insecticide [97]. Knowing the virus strain involved in an outbreak is important in choosing an effective vaccine, the only certain way of controlling epidemics [91].

## 3. Materials and Methods

We conducted a literature search using Google Scholar and PubMed (http://www.ncbi.nlm.nih.gov) using the terms “arboviruses” or “vector”. The keywords used were “arboviruses AND Thailand”. The timeframe was not specified, therefore accessing all records available from the two search platforms used. The titles and abstracts of the search results were then examined for relevance, and appropriate ones were examined in more detail. The mosquito vectors and collections were recorded in Thailand from 2009–2019 is shown in Table 1.

## 4. Conclusions

This review describes the important human and veterinary arboviruses, their vectors and potential vectors, comprising mosquito species, sand flies, ticks, and cimicids in Thailand. Most arbovirus vectors in Thailand are *Aedes* spp. mosquitoes. They transmit dengue, chikungunya, and Zika viruses. *Ae. aegypti* is the major vector of dengue and Zika virus transmission, while *Ae. albopictus* is the major vector of the chikungunya virus in Thailand [14,24,29]. Both of *Ae. aegypti* and *Ae. albopictus* are co-circulated in Thailand. Moreover, coinfection also occurs between two genotypes of the dengue virus; DENV-2 co-infects with DENV-3 and DENV-3 also co-infects with DENV-4 in larvae of *Ae. albopictus* [106]. DENV-5 is typically transmitted by the sylvatic cycle, unlike the other four serotypes [107]. Nowadays, there is no indication that DENV-5 is present in Thailand, although the vector is present.

In Thailand, emerging viral diseases have been reported annually, especially insect-borne diseases such as DF that occur every year. The heteroserotypes of the dengue virus has been found to co-circulate in *Ae. aegypti* and *Ae. albopictus* in natural populations [24]. Chikungunya virus also has been detected in both these mosquito vectors captured from the field during a large outbreak of chikungunya fever in 2009 [24,29]. Laboratory studies of dengue and chikungunya virus coinfection in *Ae. aegypti* mosquitoes in Malaysia showed them to be refractory to coinfection [27], while the *Ae. albopictus* C6/36 cell line showed competitive suppression during coinfection of DENV-3 and CHIKV, respectively [28]. Studying vector competence is important to gain knowledge about the virus-vector relationship. Since 2002, the DENV strain (633798) that isolates from patients in Thailand in 1963 has been inoculated to *Ae. aegypti*. These studies reported the persistence of the dengue virus in *Ae. aegypti* until the seventh generation under laboratory conditions [108]. Furthermore, natural transovarial transmission (TOT) of the dengue virus has been detected in both adult males and females of pale and dark form *Ae. aegypti* mosquito in Thailand [34]. Not only TOT of dengue virus has been reported, the vertical transmission of the Zika virus in *Cx. quinquefasciatus* and *Ae. aegypti* have been recorded. The female *Cx. quinquefasciatus* are able to transmit the Zika virus to their progeny until the sixth generation, while males are able to transmit the Zika virus until the second generation [50]. On the other hand, the Zika virus could be detected in the progeny of *Ae. aegypti* until the F1 generation, while *Ae. albopictus* has not been transmitted to offspring in the laboratory [50]. Understanding the mosquito species involved is very important for vector control and public health.

Many arthropod vectors in Thailand, then viral disease prevention and vector control strategies need to be supported as an integral part of public health management. Surveillance and access to outbreak data are very important to protect communities. Rapid detection of arbovirus infections in humans, wildlife or livestock, may be critical to resolving the emergence of such arboviruses in the future [109].

## Figures and Tables

**Table 1:** Important mosquito vectors and non-vectors reported in Thailand 2009–2019.

Vector Species *	Virus Transmission ***	Collected Sample **	References
*Aedeomyia catasticta*		LTs	[16]
*Aedes aegypti*	DENV, CHIKV, ZIKV, Phleboviruses	LTs, GTs, LC, HLC	[16,17,18,19]
*Aedes albopictus* (Skuse)	DENV, CHIKV, ZIKV	LTs, GTs, HLC	[13,17,18,19]
*Aedes lineatopennis*		LTs	[13]
*Aedes mediolineatus*		LTs	[16]
*Aedes scutellaris* (Walker)	DENV, CHIKV	LC	[17]
*Aedes vexans*		LTs	[16]
*Anopheles argyropus*		LTs	[16]
*Anopheles barbirostris*		LTs	[13,19,20]
*Anopheles baimaii*		CBC, HLC	[21]
*Anopheles campestris*		LTs	[16]
*Anopheles dirus*		CBC, HLC	[21]
*Anopheles epiroticus*		HLC	[18]
*Anopheles kochi*		LTs	[13,20]
*Anopheles minimus s.l. Theobald,*		LTs	[20,22]
*Anopheles maculatus s.l. Theobald*		LTs	[20,22]
*Anopheles nigerrimus*		LTs	[16]
*Anopheles peditaeniatus*		LTs, GTs	[16]
*Anopheles sinensis*		LTs	[16]
*Anopheles stephensi*		LTs	[13]
*Anopheles subpictus*		LTs	[19]
*Anopheles sundaicus*		LTs, GTs	[16]
*Anopheles tessellatus*		LTs	[13,16,20]
*Anopheles vagus*		LTs, GTs	[16]
*Armigeres subalbatus*		LTs, GTs	[13,16,20]
*Coquillettidia crassipes*		LTs	[13,16]
*Culex bitaeniorhynchus*		LTs, GTs	[13,16]
*Culex fascocephala*		LTs	[13,16,20]
*Culex gelidus*	TMUV	LTs, GTs	[13,16]
*Culex pseudovishnui*		LTs, GTs	[16,20]
*Culex quinquefasciatus*	ZIKV, JEV, TMUV	LTs, GTs, HLC	[13,16,18,19,20,23]
*Culex sitiens*		LTs, GTs, HLC	[16,18]
*Culex tritaeniorhynchus*	TMUV	LTs, GTs	[13,16]
*Culex vishnui*	TMUV	LTs, GTs	[13,16,19,20]
*Culex whitmorei*		LTs	[19]
*Lutzia fuscana*		GTs	[16]
*Mansonia annulata*		LTs	[13]
*Mansonia annulifera*		LTs	[13]
*Mansonia bonneae*		LC	[17]
*Mansonia indiana*		LTs, GTs	[16]
*Mansonia uniformis*		LTs	[13,16]
*Uranotaenia lateralis*		LTs	[13,20]

* The bold text indicates the mosquito species presenting with positive virus infection in Thailand. ** Collection method using light traps (LTs), gravid traps (GTs), larvae collection (LC), human landing catch technique (HLC), and cattle baited collections (CBC). *** The abbreviations of the virus are described: dengue virus (DENV), chikungunya virus (CHIKV), Zika virus (ZIKV), Japanese encephalitis virus (JEV), Tembusu virus (TMUV).

**Table 2:** Current arboviruses in Thailand and their vertebrate hosts.

Arboviruses *	Disease(s)	Vector	Vertebrate Host	Genetic Variability	References
**Genus *Flavivirus*, and family Flaviviridae**					
Dengue virus (DENV)	Dengue fever, DHF and DSS	*Aedes aegypti* and *Aedes albopictus*	Human	Four genotypes: dengue 1 to 4 (DENV 1–4)	[98,99,100]
Japanese encephalitis virus (JEV)	Japanese encephalitis	*Culex tritaeniorhynchus*	Domestic pigs, immigration birds and horse	Five genotypes	[13,53,56,101]
West Nile virus (WNV)	West Nile fever, encephalitis	*Culex* mosquitoes, especially *Cx. pipiens*, *Ae. albopictus*	Passerine, birds	Contain at least 2 lineages, isolated from different geographic	[3,61,102,103,104]
Zika virus (ZIKV)	Zika fever or Zika virus disease	*Aedes aegypti*	Monkeys	2 lineages; the African lineage and the Asian lineage	[14,50]
Tembusu virus (TMUV)	Duck egg-drop disease	*Cx. tritaeniorhynchus*, *Cx. quinquefasciatus*, *Cx. gelidus* and *Cx. vishnui*	Duck, geese, house sparrow, pigeons, mosquito and human	From duck, avian and human; DTMUV, avian TMUV, and TMUV	[7,82,83,85,105]
Tick-borne viruses (TBVs): Langat virus (LGTV)	Tick-borne encephalitis	*Haemaphysalis* and *Ixodes*	Hard tick, wildlife and human	Contain two orders, nine families and at least 12 genera	[8,64,68]
Kaeng Khoi virus (KKV)	Kaeng Khoi virus fever	*Cimicidae* such as *Stricticimex parvus* and *Cimex insuetus*	Human, bats	PSC-19 and WDBC1403	[6,87]
**Genus *Alphavirus*, and family Togaviridae**					
Chikungunya virus (CHIKV)	Chikungunya fever	*Aedes* mosquitoes, especially *Aedes aegypti* and *Aedes albopictus*	Human	East/Central/South Africa (ECSA), the Indian Ocean lineage (IOL)	[28,29,40]
**Genus *Orbivirus*, and family Reoviridae**					
African horse sickness virus (AHSV)	African horse sickness	Culicoides species; *Culicoides imicola* and *C. obsoletus*	Horse	Viral from southern Africa, UK (Spain)	[93,94]

* Arboviruses are a group of viruses that are transmitted by insects to human. The word arboviruses are an acronym (Arthropods borne viruses) disease.

## Data Availability

Data is contained within the article.
